# Advances in understanding cartilage remodeling

**DOI:** 10.12688/f1000research.6514.1

**Published:** 2015-08-28

**Authors:** Yefu Li, Lin Xu

**Affiliations:** 1 Department of Developmental Biology, Harvard School of Dental Medicine, Boston, MA, USA; 2 Faculty of Medicine, Harvard Medical School, Boston, MA, USA

**Keywords:** Cartilage remodeling, osteoarthritis, transforming growth factor-beta 1, aggrecanase, chondrocytes, chondron

## Abstract

Cartilage remodeling is currently among the most popular topics in osteoarthritis research. Remodeling includes removal of the existing cartilage and replacement by neo-cartilage. As a loss of balance between removal and replacement of articular cartilage develops (particularly, the rate of removal surpasses the rate of replacement), joints will begin to degrade. In the last few years, significant progress in molecular understanding of the cartilage remodeling process has been made. In this brief review, we focus on the discussion of some current “controversial” observations in articular cartilage degeneration: (1) the biological effect of transforming growth factor-beta 1 on developing and mature articular cartilages, (2) the question of whether aggrecanase 1 (ADAMTS4) and aggrecanase 2 (ADAMTS5) are key enzymes in articular cartilage destruction, and (3) chondrocytes versus chondron in the development of osteoarthritis. It is hoped that continued discussion and investigation will follow to better clarify these topics. Clarification will be critical for those in search of novel therapeutic targets for the treatment of osteoarthritis.

Cartilage remodeling is a continuous process in which the existing cartilage is removed (or degraded) and replaced by new cartilage (regenerated). The balance between degradation and regeneration has been considered so critical that tipping the scale toward degradation results in osteoarthritis (OA). Therefore, an understanding of the molecular basis of the degradation and regeneration processes will undoubtedly provide valuable information for those in search of novel therapeutic protocols for the treatment of OA. In this review article, we focus on several recent discoveries on the topic of articular cartilage degeneration.

With regard to the remodeling of the extracellular matrix, mature articular cartilages are, in general, considered relatively quiescent tissues. For example, a study by Verzijl *et al*. indicates that the half-life of collagens in human mature cartilages is 117 years ^1^. The long half-life of the collagens indicates a slow turnover of the collagens in the cartilages. However, the half-life of the aggrecan (the large monomer) turnover is about 3.4 years ^2^. This suggests that the rate of the turnover may be different in the different parts of the extracellular matrix. For example, the rate of the turnover is high in the aggrecan-rich pericellular matrix of chondrocytes and the rate of the turnover is low in the collagen-rich interterritorial and territorial matrices in articular cartilages ^3^.

Although this review focuses on articular cartilages, we have to point out that OA is currently considered the consequence of the whole joint failure. In addition to articular cartilages, the subchondral bone, peri-articular cartilage, synovial membrane, ligaments, and menisci contribute to the development of OA ^4^.

## Biological effect of transforming growth factor-beta 1 β on developing and mature articular cartilages

Is transforming growth factor-beta 1 (TGF-β1) a culprit or protector in the development of OA? Currently, numerous pharmaceutical companies are considering the use of TGF-β1 as a stimulant to repair damaged articular cartilage for the treatment of OA. Is this the correct choice?

TGF-β1 has been considered an anabolic factor to articular chondrocytes, based largely on results from *in vitro* and *ex vivo* experiments in which TGF-β1 can stimulate chondrocytes to synthesize and release proteoglycans and type II collagens ^5, 6^. In addition, three independent mouse genetic studies ^7– 9^ demonstrate that Tgf-β1 is required for the formation of articular cartilage at early stages of development in mice. Without Tgf-β1, articular cartilage is not formed properly, eventually an immature joint becomes an OA-like joint in mice. Moreover, a human genetic study reports that a two-nucleotide deletion, 741-742del AT (nonsense mutation), in *SMAD-3* causes early-onset OA in a human family ^10^. All of the aforementioned results support the argument that TGF-β1 is a protector against the development of OA. Unfortunately, the situation is not that simple. Numerous other independent investigations suggest that TGF-β1 may, in fact, be a factor in joint destruction. First, studies with animal models, by Itayem *et al*., suggest that intra-articular injections of TGF-β1 into adult rat knee joints cause early onset of OA ^11, 12^. Second, a human genetic study reports that a nucleotide change, 859C>T or 782C>T in *SMAD-3*, increases the level of TGF-β1 and activity of the TGF-β1 signaling pathway in human families is associated with early-onset OA ^10^. This is in agreement with the observation from two other studies indicating that the level of TGF-β1 is significantly higher in human OA tissues than in healthy articular cartilages ^13, 14^. Third, we found increases in the expression of Tgf-β1 and of p-Smad2/3 in articular chondrocytes of knee joints in mouse models of OA ^15^. The increased expression of p-Smad2/3 was associated with elevated expression of a serine protease, high-temperature requirement A1 (HtrA1), in the chondrocyte. HTRA1 is capable of degrading extracellular matrix molecules, particularly most pericellular components of articular chondrocytes ^16^. Another independent research group also demonstrates that TGF-β1 induces HTRA1 in human primary chondrocytes ^17^. Fourth, we determined whether the removal of Tgf-β type II receptor ( *Tgfbr2*) from the articular cartilage of adult knee joints could attenuate the OA progression. We deleted *Tgfbr2* in the articular cartilage of adult mouse knee joints and then subjected the mice to destabilization of the medial meniscus (DMM). We found a significant disparity in the progression of articular cartilage degeneration in knee joints between mice with or without *Tgfbr2* at 8 and 16 weeks following the surgery. The progression toward OA was significantly ( *P* <0.05) delayed in *Tgfbr2* ^−/−^ mice.

Several studies also indicate that the increase in the amount of TGF-β1 in other joint tissues has detrimental effects on adult joints. A study by Maeda *et al*. suggests that a high level of TGF-β1 does more harm than good to the tendon ^18^. One study by Bakker *et al*. reports that the constitutive overexpression of active TGF-β1 in adult mouse knee joints results in OA associated with an increase in the production of proteoglycans in articular cartilage, hyperplasia of synovium, and chondro-osteophyte formation ^19^. A study by Zhen *et al*. demonstrates that inhibition of TGF-β1 signaling in mesenchymal stem cells of subchondral bone delays the development of OA in adult mice ^20^.

How can this “conflicting” role of TGF-β1 in the pathogenesis of OA be explained? One plausible explanation is that effective TGF-β1 signaling acts in a dose-dependent manner. In this scenario, an appropriate level of TGF-β1 is required for the development and maintenance of articular cartilages. Therefore, TGF-β1 below or above this level results in articular cartilage degeneration. However, results from our study with mice without *Tgfbr2* suggest that the TGF-β1 dose-dependent manner may not be the case. Another plausible explanation is that effective TGF-β1 signaling acts in a developmental stage-dependent manner. In this scenario, TGF-β1 is required for the development of articular cartilage; however, once a joint is formed, TGF-β1 is no longer needed. In any case, induction of TGF-β1 in an adult joint causes articular cartilage degeneration. Therefore, inhibition activity of TGF-β1, not application of TGF-β1, may be considered in treatment of OA in mature joints.

## Are ADAMTS4 and ADAMTS5 key enzymes in articular cartilage destruction?

Proteoglycans are the basic elements of articular cartilage and are indispensable in the ability of articular cartilage to resist compressive pressure. Thus, much of the effort in the OA research field is focused on the search for an enzyme, or enzymes, that degrades proteoglycan. In 1999, two enzymes, ADAMTS-4 and ADAMTS-5, were cloned ^21, 22^. Both of these aggrecanases degrade aggrecans (proteoglycans). This indicates that both aggrecanases may be ideal therapeutic targets in the development of disease-modifying OA drugs. In fact, two independent research groups used mouse gene-targeting techniques to delete ADAMTS-4 or ADAMTS-5. One group found that the deletion of ADAMTS-5 could protect aggrecan from being degraded in a mouse model of inflammatory arthritis. With regard to the development of OA, it is not clear whether both aggrecanases are involved in cartilage destruction ^23^. The results from another group demonstrated that the removal of ADAMTS-5 in mice could significantly delay the progressive process of articular cartilage degeneration at 4–8 weeks following the DMM surgery ^24^. This suggests that ADAMTS-5 may play a role in early stages of OA development. However, lack of evidence indicating elevated expression of ADAMTS-5 at early stages of articular cartilage degeneration in any one of the existing mouse models of OA raises a question as to how important a role this enzyme has in the development of OA.

More importantly, a recent study indicates that the expression of ADAMTS-5 is increased in the articular cartilage of knee joints in adult mice because of inactivation of *Sox9* ^25^. The elevated expression of ADAMTS-5 is associated with the disappearance of aggrecans. Surprisingly, there is no progression of articular cartilage degeneration in this model. This is contrary to our current understanding that aggrecans are indispensable for articular cartilage health. Consistent with this observation, another independent investigation indicates that an increase in the expression of bone morphogenetic protein 2 (Bmp2) elevates levels of the neo-epitope, VDIPEN341, of aggrecan in articular cartilage without inducing an acceleration of cartilage degeneration in mice. Furthermore, Davidson *et al*. find that the increased expression of *Bmp2* does not exacerbate the degenerative condition of articular cartilage that has been induced by the DMM in mouse knee joints ^26^.

One plausible explanation for the aforementioned observation is that the loss of proteoglycans alone may not be sufficient to initiate or accelerate articular cartilage degeneration. Instead, the degradation of both proteoglycans and type II collagen may be required in the development of OA. Interestingly, a study by Karsdal *et al*. demonstrates that articular cartilage degradation is completely reversible in the presence of high levels of aggrecanase-mediated aggrecan degradation but irreversible after induction of metalloprotenase (MMP)-mediated aggrecan and collagen type II degradation ^27^. This study suggests that the aggrecanases may be involved with the reversible processes of cartilage degradation (or extracellular matrix turnover) but MMPs cause the irreversible degeneration of articular cartilages.

There is evidence that the removal of ADAMTS-5 may protect joints against OA by stabilizing subchondral bone ^28^. Thus, it will be important to understand whether aggrecanases play roles in the development of OA through other joint tissues.

We discuss the aggrecanases in this brief review. However, other enzymes, such as MMPs, elastase, and cathepsins, also play important roles in the pathogenesis of OA.

## Chondrocytes versus chondron in the development of osteoarthritis

Primary chondrocytes and chondrocyte cell lines are the primary tools for *in vitro* experiments in cartilage research and repair. They have allowed a wealth of information to be obtained about the genetic regulation of chondrocyte function, activation of signaling pathways, and gene expression profiles in chondrocyte response to chemical or mechanical stimulation. Many investigators use chondrocytes *in vitro* to study physiological and pathophysiological events while mimicking *in vivo* biological conditions. In particular, researchers almost exclusively use primary chondrocytes as a resource to regenerate functional articular cartilage. Regarding this method, however, a question remains: are primary chondrocytes alone adequate for investigating the role of chondrocytes in OA development and articular cartilage repair?

Chondrocytes and their pericellular matrix are considered to be the primary structural and functional units, termed chondrons, of articular cartilage ^29– 35^. This concept was proposed by Benninghoff in 1925. About 40 years later, Szirmai further evaluated the structure of the chondron by a more systematic analysis. At that time, however, the chondron was not widely recognized as a functional unit. Some 20 years later, C.A. Poole’s research group completed additional experiments to physically isolate chondrons from cartilage and showed that chondrons are true anatomic and functional entities. Chondrons consist of chondrocytes, the pericellular matrix, and a capsule surrounding the pericellular matrix. The pericellular matrix contains laminin, fibronectin, biglycan, decrin, fibromodulin, matrilin 3, and cartilage oligo matrix protein (COMP). The pericellular capsule is composed mostly of type VI and IX collagen and proteoglycans. The capsule and the pericellular matrix separate chondrocytes from the adjacent interterritorial or territorial matrices containing type II collagen. Clearly, under normal conditions, type II collagen is not exposed to chondrocytes. It is conceivable that disruption of the pericellular matrix exposes chondrocytes to type II collagen and can alter the metabolic events in chondrocytes, eventually leading to cartilage destruction. In fact, results from human and mouse genetic studies indicate the significant role of the pericellular matrix in protecting articular cartilage against the development of OA. For example, the deficiency of one or a combination of two components of the pericellular matrix, such as type VI collagen, type IX collagen, matrilin 3, decrin, biglycan, and fibromodulin, results in early onset of OA in mice ^36– 40^. In human genetic studies, mutations in type IX collagen and COMP are associated with OA ^41– 46^.

In 1998, a study by Lee and Loeser provided evidence that the pericellular matrix of chondrocytes could play critical roles in the maintenance of normal metabolic activities of chondrocytes in articular cartilage and that the disruption of the pericellular matrix is associated with articular cartilage degeneration ^47^. Very interestingly, the significant role of the pericellular matrix of a cell is also demonstrated in neural tissue. A study by Gogolla *et al*. points out that the perineuronal net (pericellular matrix of neurons) in the amygdala plays a significant role in protecting the neurons from the loss of “fear memory” ^48^. The authors also find that functional and structural changes of sensory systems are caused by the absence of a perineuronal net in the visual cortex. The significant role of the pericellular matrix of a neural cell in the brain coincides with its perceived role within the chondron. If that is the case, maintaining the integrity of the pericellular matrix will be one of the key issues in protection against OA. We believe that more attention should be directed toward the pericellular matrix of chondrocytes for the identification of novel biomarkers and therapeutic targets for OA.

Data from a very recent investigation provide more evidence that chondrons are not only the functional unit in the maintenance of articular cartilage homeostasis but also the basic elements in the regeneration of articular cartilage. Chondrons derived from adult articular cartilage are more efficient than chondrocytes in the regeneration of articular cartilage. This information is particularly critical for the articular cartilage repair field ^49^.

Collagen type VI is one of the major components of the capsule of the pericellular matrix. Results from one very recent study indicate that soluble collagen type VI can be a stimulant for chondrocyte proliferation. The soluble collagen type VI may also prevent proliferating chondrocytes from being dedifferentiated *in vitro* ^50^. It is well known that chondrocyte dedifferentiation is one of the major obstacles in cartilage tissue repair. A study by Zelenski *et al*. shows that the deletion of type VI collagen alters the mechanical properties of the pericellular matrix of chondrocytes ^51^. This, in turn, increases the extent of cell swelling and osmotically induced transient receptor potential cation channel subfamily V member 4 (TRPV4) signaling in an age-dependent manner. These findings suggest that alterations in pericellular matrix properties can influence mechanotransduction via TRPV4 or other ion channels, which eventually leads to articular cartilage destruction.

In summary, chondrons, instead of primary chondrocytes or chondrocyte cell lines, may be a more appropriate choice for investigating the biological functions and effects of chondrocytes in the development of OA and cartilage repair.
