# Supplementary material for: Advances in understanding cartilage remodeling
Source: F1000Res. 2015 Aug 28;4(F1000 Faculty Rev):642. [Version 1] doi: 10.12688/f1000research.6514.1 (PMC4560251; doi:10.12688/f1000research.6514.1)
Supplement: Peer review history

# Peer review: Referee response for version 1

**DOI:** 10.5256/f1000research.6990.r10170

**Published:** 2015-08-28

**Authors:** Farshid Guilak

**Affiliations:** 1 Duke University, Durham, NC, USA

I have read this submission. I believe that I have an appropriate level of expertise to confirm that it is of an acceptable scientific standard.

# Peer review: Referee response for version 1

**DOI:** 10.5256/f1000research.6990.r10169

**Published:** 2015-08-28

**Authors:** Richard Loeser

**Affiliations:** 1 University of North Carolina School of Medicine, Chapel Hill, NC, USA

I have read this submission. I believe that I have an appropriate level of expertise to confirm that it is of an acceptable scientific standard.
